# Field Evaluation of Arbuscular Mycorrhizal Fungal Colonization in *Miscanthus* × *giganteus* and Seed-Based *Miscanthus* Hybrids Grown in Heavy-Metal-Polluted Areas

**DOI:** 10.3390/plants11091216

**Published:** 2022-04-29

**Authors:** Alicja Szada-Borzyszkowska, Jacek Krzyżak, Szymon Rusinowski, Krzysztof Sitko, Marta Pogrzeba

**Affiliations:** 1 Institute for Ecology of Industrial Areas, 6 Kossutha St., 40-844 Katowice, Poland; a.szada-borzyszkowska@ietu.pl (A.S.-B.); j.krzyzak@ietu.pl (J.K.); s.rusinowski@ietu.pl (S.R.); krzysztof.sitko@us.edu.pl (K.S.); 2 Plant Ecophysiology Team, Institute of Biology, Biotechnology and Environmental Protection, University of Silesia in Katowice, 40-032 Katowice, Poland

**Keywords:** indigenous microorganisms, arbuscular mycorrhiza, phytoremediation, heavy metals, energy crops, *Miscanthus*

## Abstract

Understanding the behavior of arbuscular mycorrhizal fungi (AMF) associated with plants is essential for optimizing plant cultivation to the phytoremediation of degraded soils. The objective of the present study was to evaluate the differences in AMF root colonization between novel seed-based interspecific *Miscanthus* hybrids (*M. sacchariflorus* × *M. sinensis*) and the standard *M.* × *giganteus* when grown in soils contaminated with heavy metals (Pb, Cd, and Zn). During the third and fourth growing seasons, higher concentration of metals in the roots and a limited transfer of metals from the roots to the shoots were observed in all the plants studied. After the third growing season, the lowest values of AMF colonization rates were observed for the *GNT34* hybrid. After the fourth growing season, AMF colonization decreased, which could be due to the drought that occurred during that season. *GNT34* showed a lower tendency to develop mycorrhizal structures on heavy-metal (HM)-contaminated soils than *GNT41* and *M × g*; however, this hybrid was insensitive to changes in colonization rates during the dry growing season.

## 1. Introduction

Out of concern for the environment and human health, many techniques have been developed to remove pollutants and restore soil function. Nowadays, one of the alternative techniques used to exclude pollutants from the soil is phytoremediation, which involves the stabilization, degradation, and/or extraction of toxic elements by plants and their associated microorganisms [1,2]. Phytoremediation has attracted great interest because it offers many advantages over chemical and physical methods, including low cost, environmental friendliness, and no secondary pollution [3,4]. The selection of plant species with tolerance to heavy-metal (HM) stress, the ability to grow rapidly under unfavorable soil conditions, high biomass yields, and the ability to concentrate stored metals in tissues can significantly improve the efficiency of this process [5]. The usefulness of plants for phytoremediation has already been described in the literature [6,7]. Among perennial energy crops, many species are suitable for phytoremediation [8,9]. Biomass production from energy crop species in areas excluded from food production could be an alternative to maintaining their continuous use [10,11], and its usability has already been studied [12,13].

One of the energy crops with high potential for biomass production is the perennial rhizomatous grass *Miscanthus* spp., which has many characteristics that make it suitable for phytoremediation [14]. *Miscanthus* species, which occur naturally in East Asia over a wide climatic range, are characterized by high photosynthetic efficiency associated with the C4 photosynthetic pathway [15,16], and can grow in soils contaminated with HM and accumulate toxic elements in their tissues [17,18]. *Miscanthus* × *giganteus* (*M × g*) is a sterile triploid hybrid of the diploid *Miscanthus sinensis* and the tetraploid *Miscanthus sacchariflorus* species, and must be vegetatively propagated [19]. Seed-based cultivation of *Miscanthus* hybrids is an alternative in the European climate because *Miscanthus* hybrids may be more cold- and drought-tolerant than *M × g* [20,21]. Considering its high yields, *M × g* is a widespread species among energy crops grown in Europe [20].

To improve phytoremediation efficiency, the use of microorganisms in the rhizosphere, such as arbuscular mycorrhizal fungi (AMFs), which can support phytoremediation processes, may be a sustainable solution [22]. AMFs are found in all types of soils worldwide and can form symbiotic associations with the root systems of most plants found in terrestrial ecosystems [23]. Mycorrhizal fungi play an important role in exchanging necessary nutrients and minerals between the soil, or soil substrate, and the cells of host plants [24]. In addition, symbiosis with AMFs has a positive effect on increasing the resistance of vascular plants to drought and excessive soil salinity [25,26]. Mycorrhizal fungi can enhance plant growth and development by increasing the uptake of water and minerals and improving plant adaptation to stressors by increasing plant resistance to toxic metals [27]. In addition, AMFs can reduce the concentration of trace elements in plant tissues by diluting and accumulating HMs in the roots [28]. Selecting appropriate host plant species and understanding plant–AMF interactions is critical to optimizing the phytoremediation process. The role of AMFs in *Miscanthus* roots during the phytoremediation process has been studied, but most studies were based on experiments using an inoculum [29,30]. Previous work on the same plot showed that *M × g* roots in soils contaminated with HM were highly colonized by native AMFs [31,32]. With the knowledge of AMF colonization in the roots of *M × g* in soil contaminated with HM, and AMF characteristics, the autochthonous AMF root colonization of novel drought-resistant seed-based interspecific *Miscanthus* hybrids (*M*. *sacchariflorus* × *M*. *sinensis*) was compared with the commonly grown genotype *M × g*. In addition, this study sought to evaluate the accumulation of HMs (Pb, Cd, and Zn) by seed-based *Miscanthus* hybrids compared with *M × g*. During the field trial, the effect of AMFs on the uptake of HM was evaluated, which is rarely found in the literature. Both AMF colonization in the roots and HMs accumulation in the tissues of *Miscanthus* were evaluated after the third and fourth growing seasons. The data obtained were used to select the hybrids with the highest or lowest AMF colonization rate on HM-contaminated sites, and to select the appropriate hybrids to improve the phytoremediation process.

## 2. Materials and Methods

### 2.1. Experimental Design

**Figure 1:**
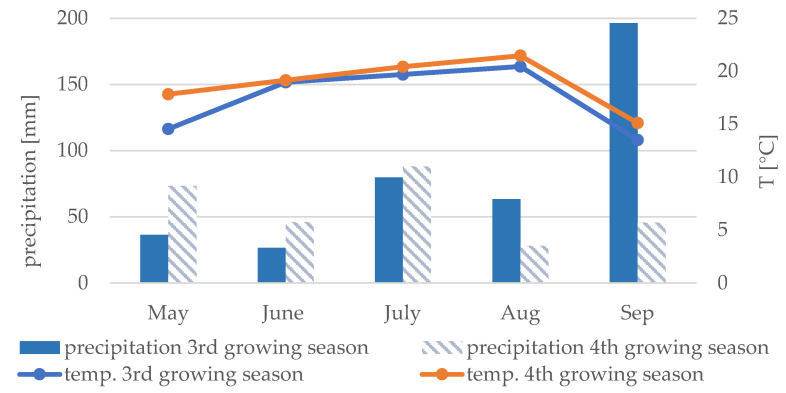
Monthly sums of precipitation and averages of temperature for Katowice, Upper Silesia in 2017 (3rd growing season) and 2018 (4th growing season).

The plantation of *Miscanthus* was established in 2015 in Bytom, Poland (50°20′41.9″ N 18°57′19.9″ E). The experimental site was located on farmland contaminated with HM as it was in close proximity to a currently abandoned lead and zinc smelter [33]. The experimental plot was divided into 15 subplots (25 m^2^). Prior to planting, soil samples were collected from each plot from a depth of 0–25 cm, to determine soil physicochemical parameters and total and bioavailable forms of HMs (Pb, Cd, and Zn). Two rhizomes of *M × g* and two novel interspecific seed-based *Miscanthus* hybrids (*GNT41* and *GNT34*) were planted per m^2^ in each experimental plot. The seed-based *Miscanthus* hybrids were bred by Aberystwyth University during the GIANT LINK program in the UK (LK0863). All plants tested were grown in triplicate. No fertilizers or inoculum were used during the experiment [10]. Towards the end of the 3rd and 4th growing seasons, the above-ground plant parts and roots were collected. Monthly sums of precipitation and averages of temperature recorded during the 3rd and 4th growing seasons are presented in Figure 1. Meteorological data were recorded in a meteorological station located in the backyard of IETU, Katowice (Upper Silesia, Poland). Average values of temperature (measured from May and September) during 3rd growing season oscillated from 14–20 °C, and in the next growing season, from 15 to 21 °C. Total precipitation recorded during 3rd growing season was about 403 mm, whereas in the next growing season, it was 281 mm.

### 2.2. Analysis of Soil Properties

Air-dried and sieved soil samples were analyzed for pH, electrical conductivity, total content, and bioavailable forms of HMs using standardized methods. Soil pH was measured in H_2_O and 1 M KCl (ratio 1:2.5 m/v) using a combined glass/calomel electrode (EPS-1, Elmetron, Zabrze, Poland) and a pH meter (CPC-505, Elmetron, Zabrze, Poland). The electrical conductivity (EC) was determined using an ESP 2ZM electrode (EUROSENSOR, Gliwice, Poland) according to the Polish standard [34], using the same instrument as for pH. The pseudo total Cd, Pb and Zn concentrations in the soil (<0.25 mm) were analyzed using flame atomic absorption spectrometry (SpektrAA 300, Varian INC., Palo Alto, USA) after hot-plate digestion in *aqua regia* [35]. Bioavailable forms of HM in soil were determined by extracting 3 g of air-dried soil with 30 mL of 0.01 M CaCl_2_ [36]. Bioavailable metal concentrations were determined in the filtrate using a flame atomic absorption spectrometer [10].

### 2.3. Plant Sampling and Sample Preparation

The plant shoots and roots were collected in three replicates. Then, the root material was divided into two parts: one part for AMF colonization evaluation and the second part for HM concentration evaluation. The shoot and root material were washed in tap water to remove surface contaminants. Fresh root samples were stained in 95% ethanol at 4 °C until microscopic slides were prepared. For analysis of Pb, Cd, and Zn concentrations, the aerial part of the plant and roots were dried at 70 °C for 72 h and then ground to a homogeneous powder.

### 2.4. Heavy Metal Content in Plant Tissues

After the 3rd and 4th growing seasons, the content of HMs in the roots and aerial parts of *Miscanthus* was analyzed. The content of metals in filtrates was determined using FAAS after digestion with HNO_3_ and HClO_4_ (4:1 *v*/*v*) (ETHOS 1, Milestone, Sorisole, Italy).

### 2.5. Calculation of Translocation and Bioconcentration Factors

During the 3rd and 4th growing season, metal translocation (TF) and bioconcentration (BCF) factors for *Miscanthus* were assessed. The TF of Pb, Cd, and Zn for *Miscanthus* was calculated from the ratio of the concentration of metals in the shoots to the concentration of these metals in the roots, according to formula [37]:

$$\mathrm{TF}=\frac{\mathrm{metal}\;\mathrm{concentration}\;\mathrm{in}\;\mathrm{the}\;\mathrm{shoots}\;\left({\mathrm{mg}\;\mathrm{kg}}^{-1}\right)\;}{\mathrm{metal}\;\mathrm{concentration}\;\mathrm{in}\;\mathrm{the}\;\mathrm{roots}\;\left({\mathrm{mg}\;\mathrm{kg}}^{-1}\;\right)}.$$

The BCF was calculated as the ratio between metal concentration in the shoots and metal concentration in the soil [37]:

$$\mathrm{BCF}=\frac{\mathrm{metal}\;\mathrm{concentration}\;\mathrm{in}\;\mathrm{the}\;\mathrm{plant}\;\mathrm{shoots}\;\left({\mathrm{mg}\;\mathrm{kg}}^{-1}\right)\;}{\mathrm{metal}\;\mathrm{concentration}\;\mathrm{in}\;\mathrm{the}\;\mathrm{soil}\;\left({\mathrm{mg}\;\mathrm{kg}}^{-1}\;\right)}.$$

### 2.6. AMF Colonization Measurement

Roots for microscopic observation were prepared according to a modified method [38], which included the following: cleaning of roots in 7% KOH solution at 80 °C for 35 min, followed by acidification in 5% lactic acid for 24 h and staining with 0.05% aniline blue in lactic acid for 24 h. Mycorrhizal colonization was evaluated using a Zeiss Axio Imager D2 microscope (Zen 2 software, Zeiss, Munich, Germany) according to the method of Trouvelot et al. (1986) [39]. During microscopic observation, mycorrhizal colonization of each fragment was evaluated on a scale of 0–5, and relative abundance of arbuscules was measured on a scale of 0–3. The parameters of mycorrhizal colonization, such as the frequency of mycorrhiza in the root system (F%), the values of relative mycorrhizal intensity (M%), and relative abundance of arbuscules (A%), were calculated using Mycocalc software [40].

### 2.7. Statistical Analysis

The results obtained were analyzed using Statistica 13.1 (Dell, Round Rock, TX, USA). Based on the normal distribution results, tests were selected for comparison between three or more independent groups (F-test for normal distribution, Kruskal–Wallis test for other-than-normal distribution). To keep the data clear, as long as the test selection algorithm resulted in an analysis of variance (ANOVA), the Fisher’s Least Significant Difference (LSD) post-hoc test was used to compare the significance between different variants. The significance level was *p* < 0.05.

## 3. Results

### 3.1. Physicochemical Soil Properties and Heavy Metal Concentration

The physical and chemical soil parameters are presented in Table 1. Soil pH was neutral, while EC had values (75.45–83.08 µS cm^−1^) that are in the typical range for agricultural soils in Poland. The pseudo-total concentrations of Pb found for plots *M × g* and *GNT41* were 4% and 13% lower, respectively, compared to *GNT34*. The pseudo-total concentration of Zn was similar in the *M × g* and *GNT41* plots, while it was about 24% higher in the *GNT34* plot. The concentration of the bioavailable form (for a review, see [36]) of Pb was below the limit of quantification in all the plots, while the concentration of Cd was similar in all plots (about 1.4 mg kg^−1^). The bioavailable forms of Zn did not differ significantly between plots *M × g* and *GNT41*, but Zn concentration was 27% higher in plot *GNT34*. 

### 3.2. Concentration of Heavy Metals in Plant Roots and Shoots

The concentration of HMs in the shoots and roots of *Miscanthus* is shown in Table 2. After the third growing season, the Pb concentration in the roots was not significantly different among the studied plants, while in the next growing season, the Pb concentration in the roots of *M × g* and *GNT34* was about 30% lower compared with *GNT41*. After the third growing season, the Pb concentration in the shoots of *GNT41* was 30% higher compared to *M × g* and *GNT34*. The lower values of Pb concentration in the shoots were observed after the fourth growing season, and no significant differences were observed among plants. 

After the third growing season, the Cd concentration in the roots of *GNT34* was 18% and 32% higher compared to *M × g* and *GNT41*, respectively. Moreover, after the fourth growing season, there was a decrease in Cd concentration in the roots of *M × g* and *GNT34*. 

Cadmium concentration was the same in the studied plants in the third growing season. After the fourth growing season, the highest Cd concentration in the shoots was observed in *M × g*, at 2.5 to 3 times higher than in the other hybrids. Lower Cd concentration in the shoots was observed in *GNT41* and *GNT34*, while it remained at the same level in *M × g*. 

Zn concentration in the shoots followed the same trend as that for Cd in the third growing season, while significantly lower values were observed for *GNT41* after the fourth growing season compared to the third. No significant differences in Zn concentration between growing seasons were observed for *M × g* and *GNT34*.

### 3.3. Heavy Metal Bioconcentration (BCF) and Translocation Factors (TF)

The values of TF and BCF are presented in Table 3. The TF for *M × g* was the same for Pb and Zn regardless of the growing season, whereas it was significantly higher for Cd in the fourth growing season than in the third. *GNT41* and *GNT34* showed the same pattern for the analyzed HMs, indicating significantly higher values for TF_Pb_ in the third growing season, while no significant differences were found between growing seasons for the other analyzed TFs. Regarding differences between varieties, significantly lower values for TF_Pb_ and TF_Zn_ were found for *M × g* in the third growing season. In addition, the same variety had significantly higher TF_Cd_ values in the fourth growing season. There were no significant differences between the TFs calculated for other growing seasons and HMs.

BCF showed significant differences between growing seasons, especially for *GNT41*. These were expressed as higher BCF_Pb_ and BCF_Zn_ in the third growing season compared to the fourth growing season. In addition, *GNT34* showed significantly higher BCF_Cd_ in the third growing season compared to the fourth growing season. No significant differences were observed among species for BCF_Pb_, BCF_Cd_, and BCF_Zn_ in the fourth, third, and fourth growing seasons, respectively. BCF_Pb_, in the third growing season, was highest for *GNT41*, while BCF_Cd_ in the fourth growing season was highest for *M × g*. In addition, BCF_Zn_, in the third growing season, was lowest at *GNT34*. There were no significant differences in BCFs values among the other varieties in each case described.

### 3.4. Mycorrhizal Studies

**Figure 2:**
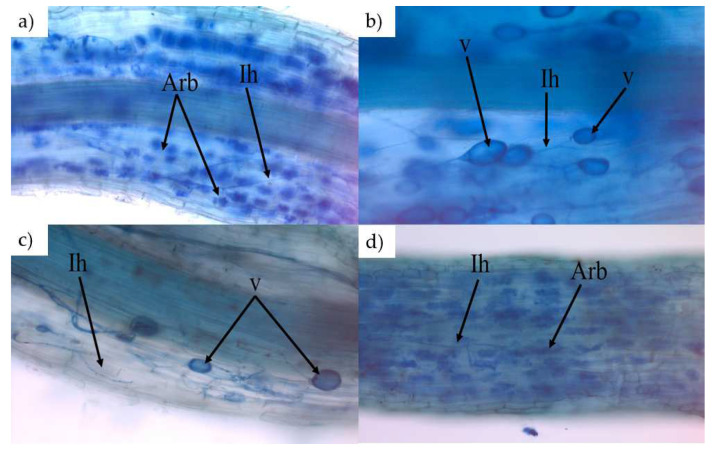
Mycorrhizal structures observed in the roots of *Miscanthus* x *giganteus* (**a**,**b**), *GNT34* (**c**) and *GNT43* (**d**) cultivated in soil contaminated with heavy metals. Structures characteristic of arbuscular mycorrhiza fungi are indicated using following abbreviations: Arb (arbuscules), v (vesicle), Ih (intercellular hyphae).

Microscopic observations showed that all the root samples of *Miscanthus* were colonized by AM fungi. Structures characteristic of arbuscular mycorrhiza, such as arbuscules (Arb), intracellular hyphae (Ih), and vesicles (v), were found in all the examined plant roots (Figure 2). 

**Figure 3:**
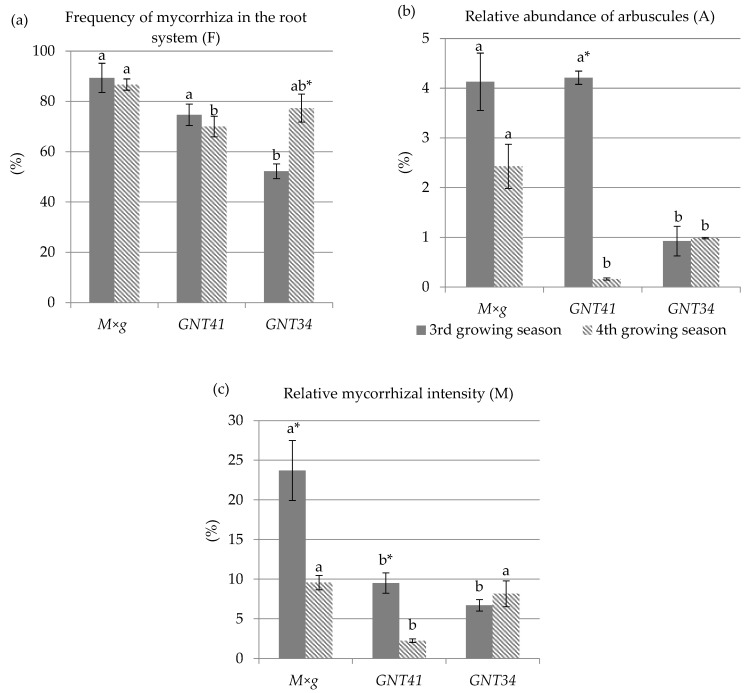
Parameters of mycorrhizal colonization in Miscanthus roots: (**a**) Frequency of mycorrhiza in the root system (F), (**b**) Relative abundance of arbuscules (A), (**c**) Relative mycorrhizal intensity (M). Values are means, ±E, *n* = 6. Asterisk (*) denotes significantly higher value of parameters, while consid-ering growing season within one hybrid. Lower-case letters (**a**,**b**) denote significant differences between different hybrids separately for both growing seasons, according to Fisher LSD test (*p* ≤ 0.05).

The values of the parameters of mycorrhizal colonization are shown in Figure 3. The results show that the highest values of mycorrhizal colonization parameters were observed among the tested plants in the roots of *M × g* after both growing seasons. After the third growing season, the values of F and A parameters for *M × g* and *GNT41* were significantly different from those for *GNT34*. The value of F for the roots of *GNT34* was lower by 41% and 30% compared to *M × g* and *GNT41*, respectively. The value of A for the roots of *GNT34* was lower by 78% compared with *M × g* and *GNT41*. The value of M for the roots of *M × g* was 2.5 and 3.5 times higher than in *GNT41* and *GNT34*, respectively. After the fourth growing season, the values of mycorrhizal colonization parameters in the roots of *GNT41* were the lowest among the tested plants. Interestingly, the value of M in the roots of *M × g* and *GNT41* decreased significantly by 60% and 76%, respectively, after the fourth growing season. In contrast, this value increased by 22% in *GNT34*. In addition, a decrease in F and A was observed in all the studied roots except *GNT34* after the fourth growing season. After the fourth growing season, the value of F and A was higher in *GNT34* roots by 45% and 7%, respectively.

## 4. Discussion

The permissible HM content in the soil exceeded the limits for the top layer (0–30 cm) of the soil on arable land according to the Polish regulation [41]. The content of pseudo-total Pb in the soil exceeded the specified limits by 6–7 times, while the contents of Cd and Zn were 11.5–13 times and 8.5–12 times higher, respectively, making the soil unsuitable for food production. A high level of heavy metals can affect the photosynthesis and the respiration of plants [42]. For example, high levels of Cd in the soil can inhibit plant growth, reduce photosynthetic activity, or cause lethal effects [43]. The studies showed that *Miscanthus* tolerates soil Cd levels of 150 mg/kg [44]. The biomass yield of *M × g* and the hybrids reached about 16–17 t ha^−1^ d.m. after the third growing season and 20–24 t ha^−1^ d.m. after the fourth growing season (unpublished data) and was comparable to the yields recorded for *M × g* in Europe in the third and subsequent years (10–25 t ha^−1^ d.m.) [45]. In addition, the effect of increasing plantation maturity was more pronounced in the case of this experiment than the water deficit reported for the fourth season. 

Some reports indicate the great ability of *Miscanthus* to absorb HMs from the soil [46]. However, the hybrids might have a different capacity for HM uptake [47,48]. This differential ability was confirmed in these studies by the different HM contents in the shoots and the roots (Table 2). The inter-seasonal observations of heavy metal content indicate the adverse effects of water deficit on the uptake of metals into the roots of *M × g* and *GNT34*. Adverse changes in the uptake of metals, nutrients, or metabolic processes and photosynthetic assimilation may be the result of stress due to water deficit [49]. Water deficit resulted in a reduction in the uptake of metals, mainly Pb and Cd, into the roots by the above *Miscanthus* genotypes by an average of 30% compared to the third growing season. This phenomenon was not observed in *GNT41*, where water deficit did not significantly affect the concentration of metals taken up into the roots. Water deficit changes in soil microbial mineralization and organic matter could limit the amount of HM available [50] and inhibit its uptake by the roots. The content of most metals such as Cu, Mn, Ni, Cd, and Pb remained constant in plants where water deficit occurred [49]. Another study showed that water deficit had a negative effect on the ability of castor bean (*Ricinus communis*) to accumulate Cd [51]. This was related to the changes in root morphology due to water deficit, which limited the uptake of Cd into the roots. This was also confirmed by studies wherein water deficit limited Cd uptake by groundnut (*Arachis hypogaea* L.) [52] and *Brassica juncea* [53]. On the other hand, studies showed that water deficit increased Cd uptake and accumulation in the shoot, root and grain of wheat [54].

After the drought period, the uptake of Cd and Zn into the above-ground parts was lower in seed-based hybrids compared to the third growing season, while no negative effect of water deficit on the uptake of the mentioned metals by *M × g* was observed. As a result, seed-based hybrids took up less Cd and Zn after the drought than *M × g*, whose heavy metal uptake ability was constant. 

The effect of heavy metal concentration in the soil on transpiration was previously reported in a pot experiment [55]. However, there is a scarcity of articles demonstrating the combined stress effect on *Miscanthus* plants. Rusinowski et al. [47] demonstrated a higher capacity for uptake of heavy metals, particularly Pb and Cd, by *M × g* compared to seed-based hybrids. This was related to differences in stomatal response time to drought stress and temperature, a critical factor in plant water strategy. Despite water availability between seasons, *M × g* took up the same amount of heavy metal, in contrast to seed-based hybrids, except Pb, which tended to behave unexpectedly [47].

The effectiveness of *Miscanthus* for phytostabilization was confirmed using computational parameters such as TF and BCF, which are widely used to evaluate the efficiency of phytoremediation processes [56]. The TF reflects the relationship between the concentration of metals in the shoots and the concentration of the respective element in the roots [57] (Table 2 and Table 3). *Miscanthus* took up less HM in the aerial parts compared to the roots (Table 2), which was reflected in the TF value of TF (Table 3). The value was less than 1, indicating a low ability to uptake metals into the aerial parts [58]; in the case of *M × g* and seed-based hybrids, this shows their potential property to exclude HMs and makes this plant a potential candidate for the phytostabilization process of the mentioned HMs [37]. These results are confirmed by previous reports [55,59]. In phytostabilization experiments, the TF ratio should be as low as possible [60,61,62]. The BCF was defined as the ratio of metal (loid) content in the shoots to that in the soil, and it is helpful in assessing the effectiveness of the uptake of metals from the soil to the shoots [63,64]. Similarly, as in the case of TF, all of the investigated plants reached BCF < 1 (Table 3), which confirmed that metal transfer from the soil is limited; moreover, *Miscanthus* has low potential for accumulating metals in its tissues effectively and to additionally support its suitability for the phytostabilization process [64,65,66]. 

The TF and BCF varied from plant to plant and from season to season. *M × g* translocated and bioconcentrated Cd more effectively to the shoots than seed-based hybrids. During the dry season, Pb translocation decreased in seed-based hybrids. Bioconcentration also decreased in *GNT41* (Pb and Zn) and *GNT34* (Cd) (Table 3). Water deficit can affect the mineralization process and reduce the transport of ions to the roots and shoots [49]. Interestingly, in *M × g*, the translocation of Cd increased during the drought period, while the translocation and bioconcentration of Pb and Zn were constant regardless of water status. This may be related to transpiration rates in the case of seed-based hybrids, as transpiration rates are limited during the dry period compared to *M × g* [47].

Despite the high content of HM in the soil, the structures characteristic of AMF, such as arbuscules, hyphae, and vesicles, were observed in all the roots after both growing seasons (Figure 2). The occurrence of AMFs in contaminated soils might be related to their adaptation to metal contamination [18,29,30]. In addition, the roots of *Miscanthus* were highly colonized by mycorrhizal fungi in both growing seasons, with the frequency of colonization (F%) ranging from 50% to 95% (Figure 3). Studies conducted after the second growing season of *M × g* in soils contaminated with HM showed 74% lower overall colonization by native AMFs compared to uncontaminated soils [18]. Additionally, other studies [31,33] showed that *M × g* roots cultured in highly HM-contaminated soils (17 mg kg^−1^ Cd, 411–480 mg kg^−1^ Pb, and 1617–1994 mg kg^−1^ Zn) were 50–60% colonized by native AMFs after the first growing season, but the F% of these colonization values was calculated using different methods. Nsanganwimana et al. (2015) [29] showed that colonization of *M × g* in Pb-, Cd-, and Zn-contaminated soils was 36.2% in non-inoculated *Miscanthus* after the second year of cultivation. Sarkar (2015) [30] showed *M*. *sacchariflorus* root colonization of about 23% in a potted crop.

The soil was homogeneous between plots in terms of contamination, so the influence of metals on the differences in mycorrhizal colonization among the species tested could initially be excluded. An influence of the age of the hybrids on AMF colonization could also be excluded due to the same planting date [67,68]. The values of mycorrhizal colonization parameters differed significantly among *Miscanthus* plant cultivars (Figure 3). Research confirms that colonization by AMFs can differ among host plants and even among their cultivars [69,70], even in the case of *M × g* cultivars grown under the same conditions [29]. Chatzistathis et al. (2013) [70] suggested that host-plant physiology, growth parameters, and also, differences in nutrient uptake effectiveness play an important role in AMF colonization. In addition, soil water deficit leads to a decrease in available phosphorus, which may directly affect colonization [71]. Therefore, future studies on the P uptake capacity of *Miscanthus* and the P concentration in soil are necessary to evaluate the differences in colonization. *M × g* was better colonized by AMFs than seed-based hybrids after both growing seasons. In addition, a higher number of arbuscules was observed in *M × g* compared to other seed-based hybrids (Figure 3).

One of the stressors that could affect AMF community structure and composition is the occurrence of different environmental conditions between the two growing season [71]. Different responses have been observed in the research reports on the AMF response to short- or long-term water deficit. This factor may cause plants to show an increasing or decreasing trend in the percentage AMF root colonization [72,73,74,75]. These two phenomena were confirmed in this study, as the water deficit that occurred in the fourth growing season resulted in different responses of *Miscanthus* cultivars in colonizing the roots of different plants. The differential response of AMF root colonization to water deficit also depends on the plant species [73], which is characterized by different physiological responses to stress related to water deficiency [76]. The decrease in AMF root colonization rate in the roots of *GNT41* and *M × g* was observed in the fourth growing season. In the same period, the values of F% increased in *GNT34*, while M% and A% remained constant. Therefore, these data may indicate that *GNT34* has a higher tolerance to stress related to water deficiency than other hybrids. However, future research is needed to determine the taxonomic differences in AMFs in *Miscanthus* roots to identify specific responses to biotic and abiotic stresses [77].

High root colonization by AMFs with a higher content of HM, especially in the roots, might indicate that the presence of AMFs could support the stabilization/immobilization process [61,78]. The higher AMF colonization of *M × g* roots corresponded with the lowest metal translocation to the shoots and the highest Pb and Zn concentration in the roots, compared to seed-based hybrids, in which lower AMF root colonization was observed. The data obtained are in agreement with the studies [79,80], according to which mycorrhizal symbiosis increases metal uptake by the roots while decreasing uptake concentration in the shoots. In contrast, studies showed increased HM uptake into the shoots of *Micanthus* after AMF inoculation by the *Glomus* species [29]. Despite the fact that the roots of *GNT34* were less colonized with AMFs than those of *GNT43*, they were characterized by a greater ability to take up HMs into their roots. Several effects of changes in root colonization rates among hybrids were observed in relation to changes in HM metal concentrations in the roots and shoots. Along with the decrease in AMF root colonization for *M × g*, a decrease in the concentration of heavy metals in the roots was observed. Similarly, *GNT41* showed lower uptake of heavy metals into the shoots in the fourth growth period compared to the third growth period, which might be related to lower AMF root colonization. Interestingly, the number of arbuscules was significantly lower, which was related to a lower uptake of heavy metals into the shoots. The different results in seed hybrids and *M × g* may suggest that the effect of AMFs on the uptake of HM may depend on the plant and/or cultivar [29,81].

More detailed studies on the relationships between AMFs and *Miscanthus* seeds are needed to explain the mechanism of colonization and its influence on the uptake of metals in the mentioned plants.

## 5. Conclusions

The present study showed that HMs accumulate mainly in the roots of *Miscanthus*, regardless of the hybrid plant studied, which was expected since this species is not an HM hyperaccumulator. Nevertheless, the studied *Miscanthus* seed-based hybrids and M × g showed that they were suitable for the phytostabilization of heavy metals. *GNT34* showed a lower tendency to develop mycorrhizal structures than *GNT41* and *M* × *g* in HM-contaminated soils; however, this hybrid was insensitive to changes in the colonization rate during the dry growing season. Water deficits affected AMF root colonization parameters differently among the *Miscanthus* hybrids studied. *GNT34* exhibited higher stress tolerance to water deficit compared to the other hybrids. AMFs could be an important factor in the resistance of *Miscanthus* hybrids to drought stress, but further studies are needed.

## Figures and Tables

**Table 1:** Soil physicochemical parameters and heavy metal concentration at the investigated plots before *Miscanthus* plantation.

	Genotype		
	*M × g*	*GNT41*	*GNT34*
Physicochemical Soil Parameters			
pH (H_2_O)	6.50 ± 0.02 a	6.52 ± 0.04 a	6.54 ± 0.06 a
pH (KCl)	5.99 ± 0.02 a	5.99 ± 0.04 a	6.03 ± 0.04 a
EC (µS cm^−1^)	83.08 ± 0.26 a	80.90 ± 3.19 a	75.45 ± 1.94 a
Heavy Metal Concentration in Soil			
Pb (mg kg^−1^)	689.50 ± 25.20 ab	621.36 ± 3.56 b	719.63 ± 20.47 a
Cd (mg kg^−1^)	26.5 ± 1.93 a	23.17 ± 0.53 a	26.50 ± 0.66 a
Zn (mg kg^−1^)	2805.4 ± 37.6 b	2590 ± 47.1 b	3568.9 ± 80.8 a
Bioavailable Forms of Heavy Metals in Soil			
Pb (mg kg^−1^)	LOQ	LOQ	LOQ
Cd (mg kg^−1^)	1.48 ± 0.06 a	1.23 ± 0.03 a	1.57 ± 0.15 a
Zn (mg kg^−1^)	96.95 ± 0.71 b	86.59 ± 2.89 b	127.75 ± 3.45 a

Values are mean ± SE, *n* = 3. Lower-case letters (a, b) denote significant differences between means in a row, according to Fisher LSD test (*p* ≤ 0.05). LOQ—limit of quantification.

**Table 2:** Content of HM in the shoots and roots of *Miscanthus*.

	Concentration of HM					
	Pb (mg kg ^−1^)		Cd (mg kg ^−1^)		Zn (mg kg ^−1^)	
	3rd Growing Season	4th Growing Season	3rd Growing Season	4th Growing Season	3rd Growing Season	4th Growing Season
Root						
*M × g*	71.19 ± 7.84 a	48.17 ± 3.79 b	43.94 ± 2.2 ab *	25.37 ± 2.10 a	1534.26 ± 163 a	1033.8 ± 54.48 a
*GNT41*	41.46 ± 7.92 a	64.63 ± 2.08 a	36.29 ± 2.59 b	32.61 ± 0.64 a	1243.21 ± 90.6 a	946.1 ± 65.52 a
*GNT34*	61.44 ± 14.90 a	41.72 ± 0.65 b	53.60 ± 2.32 a *	31.2 ± 2.35 a	1065.6 ± 59.7 a	1123.6 ± 108.2 a
Shoot						
*M × g*	11.92 ± 0.89 b *	7.46 ± 0.97 a	0.66 ± 0.06 a	0.70 ± 0.04 a	135.1 ± 5.29 a	123.95 ± 5.18 a
*GNT41*	17.03 ± 0.45 a *	7.70 ± 0.64 a	0.52 ± 0.05 a *	0.31 ± 0.04 b	154.0 ± 9.64 a *	103.97 ± 4.16 b
*GNT34*	11.92 ± 0.89 b *	6.02 ± 0.19 a	0.66 ± 0.07 a *	0.26 ± 0.03 b	133.1 ± 5.98 a	140.25 ± 7.81 a

Values are mean, ±SE, *n* = 3. Asterisk (*) denotes significant differences between metals concentrations, when considering growing seasons within one hybrid. Lower-case letters (a, b) denote significant differences between different hybrids separately for both growing seasons, according to Fisher LSD test (*p* ≤ 0.05).

**Table 3:** Translocation and bioconcentration efficiency of heavy metals in plants.

Translocation Factor						
	TF_Pb_		TF_Cd_		TF_Zn_	
	3rd growing season	4th growing season	3rd growing season	4th growing season	3rd growing season	4th growing season
*M × g*	0.17 ± 0.02 b	0.16 ± 0.03 a	0.02 ± 0.0 a	0.03 ± 0.0 a*	0.09 ± 0.01 b	0.12 ± 0.01 a
*GNT41*	0.33 ± 0.0 a*	0.12 ± 0.02 a	0.01 ± 0.0a	0.01 ± 0.0 b	0.12 ± 0.0 a	0.11 ± 0.01 a
*GNT34*	0.25 ± 0.04 a*	0.14 ± 0.01 a	0.01 ± 0.0 a	0.01 ± 0.0 b	0.13 ± 0.01 a	0.13 ± 0.01 a
Bioconcentration factor						
	BCF_Pb_		BCF_Cd_		BCF_Zn_	
	3rd growing season	4th growing season	3rd growing season	4th growing season	3rd growing season	4th growing season
*M × g*	0.02 ± 0.0 b	0.01 ± 0.0 a	0.03 ± 0.0 a	0.03 ± 0.0 a	0.05 ± 0.0 a	0.04 ± 0.0 a
*GNT41*	0.03 ± 0.0 a*	0.01 ± 0.0 a	0.02 ± 0.0 a	0.01 ± 0.0 b	0.06 ± 0.01 a*	0.04 ± 0.0 a
*GNT34*	0.02 ± 0.0 b	0.01 ± 0.0 a	0.03 ± 0.0 a*	0.01 ± 0.0 b	0.04 ± 0.0 b	0.04 ± 0.0 a

Values are mean, ±SE, *n* = 3. Asterisk (*) denotes significantly higher value of translocation or bioconcentration factor, while considering growing season within one hybrid. Lower-case letters (a, b) denote significant differences between different hybrids separately for each growing season, according to Fisher LSD test (*p* ≤ 0.05).

## Data Availability

Not applicable.
